# A Self-Catalytic Bio-Platform for Upcycling of PET Plastic into Oligoesters for Polyurethane Synthesis

**DOI:** 10.3390/ma19142977

**Published:** 2026-07-10

**Authors:** Anjie Qi, Yunjia Liang, Bingjie Ge, Guodong Jiang, Shanglin Xiang, Dongyu Cai

**Affiliations:** 1 College of Materials Science & Engineering, Nanjing Tech University, 30 South PuZhu Road, Nanjing 211816, China; k1451495837@163.com (A.Q.); k740036134@126.com (B.G.); xiangsl@njtech.edu.cn (S.X.); 2 Key Laboratory of Flexible Electronics and Institute of Advanced Materials, School of Flexible Electronics (Future Technologies), Nanjing Tech University, 30 South PuZhu Road, Nanjing 211816, China; 18652576298@163.com

**Keywords:** castor oil, subcritical water, upcycling, PET

## Abstract

**Highlights:**

- Developed a self-catalytic water/castor oil system for PET depolymerization.
- Achieved spontaneous precipitation of oligoesters without cooling crystallization.
- Upcycled the oligoesters into polyurethane adhesives with high peel strength.

**Abstract:**

This study presents a green approach for polyethylene terephthalate (PET) upcycling using a biphasic system of subcritical water and castor oil. This system enables efficient conversion without an external catalyst and facilitates product separation. Hydrolysis of castor oil generates fatty acids in situ, which catalyze PET conversion to selectively produce low-molecular-weight oligoesters (Mn ≈ 1500 g/mol). These oligoesters are inherently immiscible with the bio-medium at room temperature, allowing straightforward separation by centrifugation. Orthogonal experiments show that temperature is the dominant factor affecting PET conversion, with the optimal conditions being 200 °C, a water-to-oil mass ratio of 1:5, and a reaction time of 10 h for complete conversion. Under the practical optimum condition, the castor oil phase remained highly effective over five consecutive depolymerization cycles. The functionalized oligoesters were used in polyurethane synthesis. At 6% loading, they gave adhesives with a T-peel strength of 8.09 N/15 mm and tensile strength of 25.14 MPa, and excellent damp-heat stability (only 0.62% loss in T-peel and 0.47% in 180° peel after aging). Thermogravimetric analysis confirmed enhanced thermal stability, with increases of 19.52 °C in T_5%_ and 16.50 °C in T_50%_ compared with the unmodified system. These results demonstrate the practical potential of the obtained oligoesters in high value adhesive applications.

## 1. Introduction

Polyethylene terephthalate (PET) is the fourth most widely produced plastic globally, and its recycling represents an urgent industrial and environmental priority [1,2]. While physical recycling yields downcycled materials with diminished quality and increased impurities, restricting their reuse in high-value applications [3]. Chemical recycling of PET aims to depolymerize waste into reusable monomers or intermediates. Early approaches were dominated by hydrolysis, which cleaves ester bonds using water under high temperature and pressure or with strong acids/bases to yield terephthalic acid and ethylene glycol [4,5,6]. Although effective, this method requires harsh conditions, high energy input, and poses challenges such as equipment corrosion and environmental concerns. Similarly, methanolysis, a form of alcoholysis, simplifies purification but still relies on toxic methanol and pressurized systems [7,8]. This has spurred the development of milder, more sustainable methods [9], notably the industrial adoption of glycolysis [10,11,12,13]. Operating under relatively moderate conditions, glycolysis using ethylene glycol produces bis(2-hydroxyethyl) terephthalate (BHET), which can be directly repolymerized into virgin-quality PET [14,15,16]. Further advances, such as aminolysis [17,18] and glycolysis [19,20] with other polyols, continue to expand routes for low-energy upcycling of PET into higher-value chemicals—reflecting an ongoing trend toward versatile, efficient, and sustainable circular economy solutions [21]. Compared with conventional glycolysis, the present strategy follows a different upcycling logic. Glycolysis mainly targets BHET for closed-loop PET re-polymerization, whereas our system is designed to directly generate oligomeric products for subsequent polyurethane synthesis.

Within this context, bio-based media offer a promising route to enhance the green profile of recycling [22]. Castor oil is a standout candidate: a fully renewable, biodegradable, and low-toxicity plant oil rich in ricinoleic acid triglycerides [23]. Its unique structure provides hydroxyl groups and a long hydrocarbon chain, which can facilitate interaction with PET. However, we found that pure castor oil alone cannot depolymerize PET. This may be attributed to the lower reactivity of its secondary hydroxyl groups compared to the primary alcohols in ethylene glycol.

To overcome this limitation, we designed a self-catalytic binary system of water and castor oil. Under subcritical conditions, water hydrolyzes the triglycerides in situ to generate fatty acids, which then catalyze PET depolymerization. This cascade reaction produces low-molecular-weight oligoesters. Due to inherent incompatibility with the medium, the oligoesters spontaneously precipitate upon cooling, enabling straightforward isolation. In contrast to a prior report on castor oil-mediated PET recycling [24], which employed microwave heating and zinc acetate as an external catalyst at 230–240 °C and treated castor oil hydrolysis as an undesirable side reaction, our approach operates under milder subcritical conditions (180 °C) by harnessing castor oil hydrolysis as the driving force for self-catalysis without any external catalyst, directly yielding separable oligoesters (Mn ≈ 1412 g/mol).We further demonstrate the direct use of these bio-based oligoesters as macromolecular building blocks for polyurethane synthesis. Overall, this work establishes a sustainable upcycling route that replaces petroleum-derived solvents, simplifies product separation, and advances PET recycling within a circular bio-economy framework.

## 2. Materials and Methods

### 2.1. Materials

PET resin was sourced from Sinopec Yizheng Chemical Fibre Company Limited, Yizheng, China (number-average molecular weight Mn = 12075 g/mol and intrinsic viscosity 0.83 dL/g). Castor oil, with a hydroxyl value of 164.84 mg KOH/g and an acid value of 1.63 mg KOH/g, was supplied by Shanghai Aladdin Biochemical Technology Co., Ltd., Shanghai, China. The following materials were also obtained from Shanghai Aladdin Biochemical Technology Co., Ltd., Shanghai, China: deionized water, ethanol (≥99.7%), ethyl acetate (≥99.5%), pyridine (≥99.5%), liquefied diphenylmethane diisocyanate (L-MDI, NCO content 30 wt.%), and poly(ethylene glycol) diglycidyl ether (DGEG, epoxy value 0.7–0.8 mol/100 g). Polycaprolactone diol (PCL, Mn = 2000 g/mol) was procured from Wangxin Plastics and Chemical Co., Ltd., Shanghai, China. A polyisocyanate curing agent, typically described as an adduct of trimethylolpropane (TMP) and toluene diisocyanate (TDI), was obtained from Shandong Lihe New Material Technology Co., Ltd., Zibo, China.3-Aminopropyltriethoxysilane (KH550, BR, 98%) was purchased from Shanghai Yuanye Bio-Technology Co., Ltd., Shanghai, China.

### 2.2. PET Depolymerization Procedure

PET pellets were sequentially rinsed with ethanol and vacuum-dried at 80 °C for 12 h prior to use. Unless otherwise specified, each depolymerization experiment used 3.0 g of pre-treated PET and 10.0 g of deionized water, with castor oil added to give water/castor oil mass ratios of 1:1, 1:3, or 1:5 (corresponding to 10.0/10.0 g, 10.0/30.0 g, and 10.0/50.0 g, respectively). The mixture was loaded into a high-pressure stainless steel autoclave. The autoclave used in this study has a volume of 250 mL, and the filling ratio was maintained below 28% based on the total liquid volume of the reaction mixture. The reaction was carried out under static conditions without stirring, and the internal pressure was not monitored due to the absence of a pressure gauge on the autoclave. The depolymerization reaction was carried out at temperatures ranging from 160 to 200 °C for 6–12 h under subcritical water conditions. Upon completion of the reaction, the reactor was cooled to room temperature. The unreacted PET solid was collected by filtration, thoroughly washed with ethanol, and subsequently dried at 60 °C under vacuum for 12 h.

PET conversion (*X*) was calculated according to Equation (1), where m_0_ and m_1_ are the initial and unreacted residual masses of PET, respectively. For each condition, conversion was determined from at least three parallel experiments, and the results are reported as mean ± standard deviation [25].

(1) $$X=\frac{m_{0}-m_{1}}{m_{0}}$$

A white powdery product (WPP) was separated from the aqueous phase at the bottom and dried for further characterization. The upper oil phase was incubated at 60 °C for 24 h, during which a white flocculent product (WFP) gradually precipitated. The resulting precipitate was washed several times with ethanol, collected by centrifugation, and dried at 60 °C for 24 h. Prior to weighing, all recovered products were thoroughly washed with ethanol to remove any surface-adsorbed oligomers or castor oil residues, ensuring the accuracy of the mass-based conversion calculation.

### 2.3. Modification of Oligoesters by Poly(ethylene glycol) Diglycidyl Ether

The reaction was conducted in a 250 mL three-necked flask fitted with a condenser, a stirrer, and a thermometer. Unless otherwise specified, a 1:30 mass ratio of oligoester to DGEG (0.8:24.0 g) was used. The mixture was then stirred under reflux at 180–200 °C for 3 h, yielding a homogeneous transparent yellow solution of DGEG-modified oligoester.

### 2.4. Synthesis of Polyurethane Adhesives

Polyurethane adhesives were synthesized in a 250 mL three-necked flask equipped with a condenser, mechanical stirrer, and thermometer. PCL, L-MDI, and ethyl acetate were first charged into the reactor at a mass ratio of 40:7:10 and reacted under reflux at 85–95 °C for 2 h to form an NCO-terminated prepolymer. Based on the PCL feed and the NCO content of L-MDI, the initial NCO/OH ratio in the prepolymer stage was 1.25, yielding an isocyanate index of 125. Subsequently, BDO was used as the conventional short-chain chain extender in the baseline formulation, while DGEG or DGEG-modified oligoester was introduced as an additional hydroxyl-containing reactive component according to the designed feed composition. It should be noted that DGEG and DGEG-modified oligoester were incorporated as reactive hydroxyl-containing co-components within the same polyurethane formulation rather than as direct one-to-one equivalent replacements for BDO. The reaction was then continued under reflux with stirring for an additional 3–5 h, yielding a transparent yellow polyurethane solution.

### 2.5. Adhesive Sample Preparation and T-Peel Test

The prepared polyurethane adhesive was homogeneously mixed with the curing agent at a mass ratio of 5:1.4 and subsequently degassed under vacuum. A uniform adhesive layer with a controlled thickness of 10 µm was applied onto a PET film using a blade coater, followed by drying in an oven at 45 °C to constant weight. The coated PET film was then laminated with aluminum foil using a hot/cold roller laminator. After curing at 45 °C for 24 h, the composite material was cut into test strips measuring 200 mm × 15 mm. The T-peel strength of these strips was evaluated at a crosshead speed of 100 mm/min in accordance with the standard GB/T 8808-1988 [26]. For each sample, at least 3 parallel laminate strips were tested for the T-peel measurement.

### 2.6. Adhesive Sample Preparation and 180° Peel Test

First, 6061 aluminum alloy sheets were cut into 200 mm × 25 mm × 1 mm specimens. The surface was wiped with a 2 wt.% KH550 ethanol solution and dried in an oven. The polyurethane adhesive and curing agent were mixed at a mass ratio of 5:1.4, followed by vacuum degassing. The adhesive was then blade-coated onto a PET film (400 mm × 25 mm × 0.1 mm) over a 200 mm × 25 mm area at a thickness of 40 μm. After drying at 45 °C to constant weight, the coated PET film was laminated onto the treated aluminum sheet using a soft roller. The assembled specimens were cured at 45 °C for 24 h, then tested at room temperature on a universal testing machine using a 180°peel configuration at a crosshead speed of 300 mm min^−1^.

### 2.7. Damp-Heat Aging Test (85 °C/85% RH)

The constant temperature and humidity chamber was set to 85 °C and 85% RH. Specimens prepared as per Section 2.5 and Section 2.6 were placed inside for 168 h. After aging, the samples were removed, equilibrated to room temperature, and tested for T-peel and 180° peel strength to evaluate the damp-heat aging resistance of the adhesive system.

### 2.8. Characterizations

The crystal structure of the samples was analyzed by X-ray diffraction (XRD, SmartLab, Rigaku, Tokyo, Japan). Surface morphology was examined using a field-emission scanning electron microscope (FE-SEM, JSM-7800F, JEOL, Tokyo, Japan). Prior to observation, the samples were sputter-coated with gold using an ion sputter coater. The SEM images were acquired at an accelerating voltage of 10 kV with a working current of 71.0 μA. Fourier-transform infrared (FTIR) spectra were recorded on a Spectrum Two spectrometer (PerkinElmer, Waltham, MA, USA) in the range of 4000–500 cm^−1^. Liquid-state nuclear magnetic resonance (^1^H NMR) spectra were obtained on an AVANCE III HD 400 MHz spectrometer (Bruker, Billerica, MA, USA) using DMSO-d_6_ as the solvent. Differential scanning calorimetry (DSC, DSC-6000, PerkinElmer, Waltham, MA, USA) and thermogravimetric analysis (TGA, HCT-1 Beijing Hengjiu, Beijing, China) were performed under nitrogen at a heating rate of 10 °C min^−1^. Gel permeation chromatography (GPC, PL-GPC 120, Agilent, Santa Clara, CA, USA) was used to determine molecular weights by using DMF as the eluent. The hydroxyl (HV) and acid values (AV) were measured following ASTM D4274-11 [27] and GB/T 12008 [28] using an automatic potentiometric titrator (WDDY-2008J, Taizhou Datang, Taizhou, China). The T-peel strength of PET/aluminum laminates was evaluated according to GB/T 8808 using a tensile testing machine (Jinan Yinuo, Jinan, China). An electric blast drying oven (DH9030A, Shanghai Jinghong, Shanghai, China), a polytetrafluoroethylene-lined reactor (HU006, Shanghai Lichen, Shanghai, China), and a programmable constant temperature and humidity chamber (XB-OTS-15013-B, Dongguan Xinbao, Dongguan, China) were used for drying, hydrothermal depolymerization, and damp-heat aging tests, respectively.

## 3. Results and Discussion

### 3.1. Chemical Structural Characterization of Oligoesters

**Figure 1:**
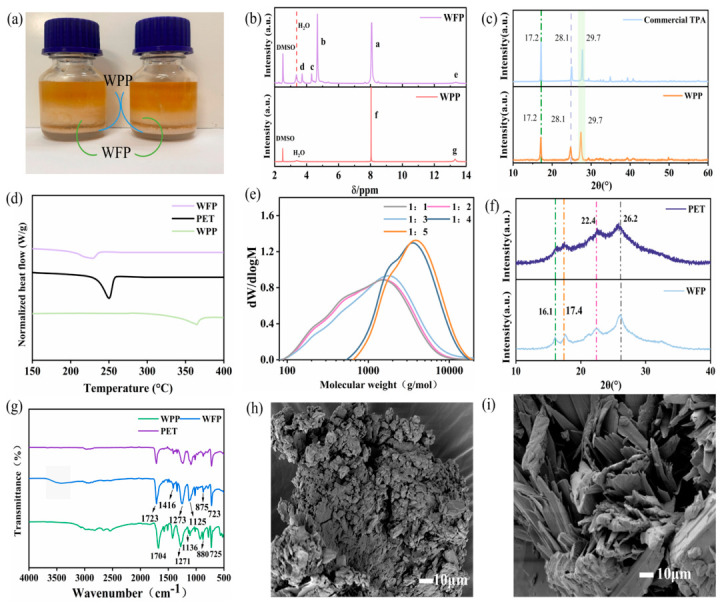
Structural characterization of PET degradation products: (**a**) Sample image of degradation products; (**b**) ^1^H NMR spectra of WPP and WFP (**c**) XRD pattern of WPP; (**d**) DSC curves of PET, WPP and WFP; (**e**) Molecular weight distribution curves of oligoesters obtained at different water-to-castor oil mass ratios (1:1 to 1:5); (**f**) XRD patterns of WFP and PET; (**g**) FTIR spectra of the degradation products; (**h**) SEM image of the WFP (×4000); (**i**) SEM image of the WPP (×4500).

The degradation process was straightforward: PET pellets were subjected to hydrothermal treatment in a biphasic mixture of water and castor oil at 180–200 °C for 10 h. Following depolymerization, the mixture spontaneously separated into distinct phases, as shown in Figure 1a. A small amount of white powdery product (WPP) precipitated in the aqueous phase at the bottom, while white flocculent product (WFP) separated from the castor oil phase and gradually accumulated at the oil–water interface over time. A series of characterizations were performed to determine the chemical composition of the degradation products. The ^1^H NMR spectrum of WPP (Figure 1b) shows a resonance at δ = 8.04 ppm (peak f), corresponding to the four aromatic protons of the benzene ring, and a peak at δ = 13.32 ppm (peak g), assigned to the carboxylic acid proton. The signals at δ = 2.51 and 3.39 ppm are attributed to residual DMSO-d6 and H_2_O, respectively. These signals are consistent with literature values [29], confirming that WPP is predominantly composed of terephthalic acid (TPA). Further evidence was provided by XRD (Figure 1c), where WPP shows characteristic diffraction peaks at 2θ = 17.2°, 28.1°, and 29.7°, in good agreement with those of commercial TPA, indicating that TPA is the dominant crystalline component in WPP [30]. The DSC curve (Figure 1d) exhibits a strong endothermic peak around 360 °C, consistent with the reported melting point of TPA [31]. Additionally, the FTIR spectrum of WPP (Figure 1g) shows characteristic absorption peaks at 1704 and 1271 cm^−1^, confirming the presence of carboxylic acid C=O and C-O bonds, while the peaks at 1136, 725, and 880 cm^−1^ are characteristic of the terephthalic acid aromatic ring [32]. Taken together, these results demonstrate that the degradation product WPP is predominantly composed of TPA.

The structural and thermal properties of the degradation product (WFP) collectively confirm its identity as low-molecular-weight oligoesters. Gel permeation chromatography (GPC) results (Figure 1e and Figure S1) show that the obtained oligoesters possess a tunable molecular weight. Figure 1e presents the normalized molecular weight distribution curves, whereas Figure S1 shows the corresponding Mn, Mw, and PDI values derived from the same GPC results. The oligoesters reach a number-average molecular weight (Mn) of 1412 g/mol with a narrow distribution (PDI = 1.48) at the optimal water-to-oil mass ratio, which is significantly lower than that of pristine PET (Mn = 12,075 g/mol) but considerably higher than that of the typical BHET monomer (Mn ≈ 254 g/mol) produced by conventional glycolysis [33]. Based on the Mn value and the molecular weight of the repeating unit of PET (192 g/mol for the terephthalate-ethylene glycol unit), the average degree of polymerization of the obtained oligoesters is estimated to be approximately 7. Crystallinity analysis further supports this conclusion. The XRD pattern (Figure 1f) exhibits broad peaks at 2θ = 22.4° and 26.2°, indicating the retention of certain crystalline features akin to PET. However, the appearance of stronger, distinct peaks at 16.1° and 17.4° suggests a restructured crystal morphology in the oligoesters. This is consistent with the DSC profile (Figure 1d), which shows a broad endothermic melting peak centered around 225 °C. This broadening can be attributed to both reduced crystallinity and the increased chain-end concentration resulting from the lower molecular weight, which together create more crystal imperfections and a more heterogeneous structure. Moreover, compared to the pristine PET’s SEM images (Figure S2, Supplementary Materials), SEM imaging (Figure 1h) shows an irregular, porous morphology of loose aggregates and fragmented flakes, consistent with polymer backbone degradation confirmed by GPC and spectroscopy. Collectively, the reduced molecular weight, altered crystallinity, and fragmented morphology indicate that WFP comprises oligoesters derived from partial PET depolymerization.

The ^1^H NMR spectrum of WFP (Figure 1b) shows a singlet at 8.10 ppm (peak a), assigned to the four equivalent aromatic protons of the benzene ring. A peak at 4.6 ppm (peak b) corresponds to hydroxyl protons, while signals at 3.7 and 4.4 ppm (peaks c and d, respectively) are attributed to the COO-CH_2_ and CH_2_-OH groups, respectively [34]. A minor signal at δ = 13.36 ppm (peak e) is ascribed to residual carboxyl end groups. The signals at δ = 2.51 and 3.30 ppm are attributed to residual DMSO-d_6_ and H_2_O, respectively. In the FTIR spectrum, the WFP exhibits a broad absorption band in the 3400–3600 cm^−1^ region, characteristic of O-H stretching vibrations. Strong bands at 1723, 1273, and 1125 cm^−1^ are attributed to ester C=O and C-O stretching modes, while the band near 1416 cm^−1^ corresponds to aromatic C=C stretching and in-plane bending. Peaks at 723 and 875 cm^−1^ are assigned to out-of-plane bending vibrations of aromatic C-H bonds [33]. These spectral features further confirm that WFP is a PET-derived oligoester product that retains structural characteristics similar to those of pristine PET.

### 3.2. Analysis of Degradation Mechanism

**Figure 2:**
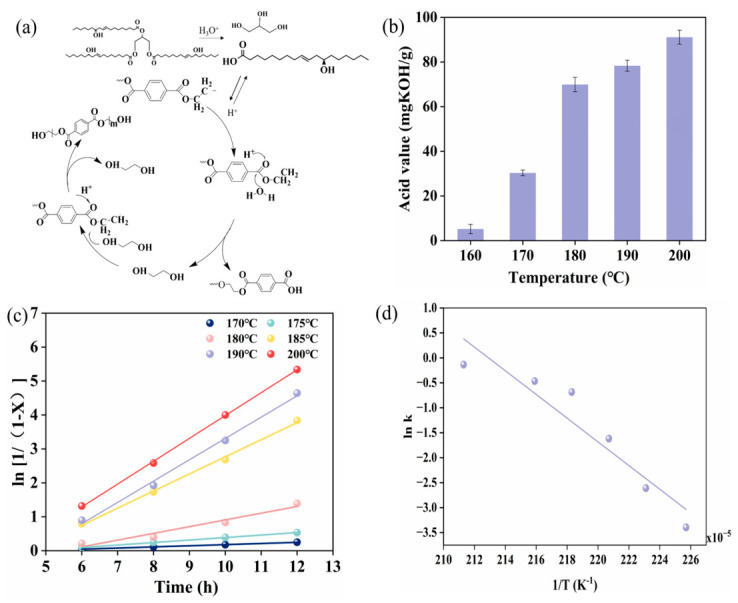
(**a**) Proposed reaction mechanism for PET depolymerization in the water-castor oil system. (**b**) Evolution of the acid value of castor oil with temperature, indicating the in situ generation of acids. (**c**) Kinetic curves fitting the PET degradation rate constants at various temperatures. (**d**) Arrhenius plot derived from the temperature-dependent rate constants for determining the activation energy.

As established in previous studies, the ionic product of water increases dramatically under subcritical conditions (from 10^−14^ to 10^−11^), resulting in significantly elevated concentrations of H^+^ and OH^−^ ions compared to ambient conditions [35]. The proposed reaction mechanism for PET depolymerization in this water-castor oil system is illustrated in Figure 2a. Initially, ester bonds in castor oil are cleaved by H_3_O^+^, generating ricinoleic acid and glycerol [36]. Under subcritical conditions, ricinoleic acid donates a proton to activate the PET carbonyl oxygen for nucleophilic attack by water, breaking the ester bond into carboxyl-terminated oligoesters and ethylene glycol. Concurrently, the released glycerol and ethylene glycol undergo alcoholysis/transesterification to generate hydroxyl-terminated oligoesters.

The variation in the acid value of castor oil with temperature is shown in Figure 2b, while the corresponding numerical data and error ranges are summarized in Table S1 in the Supplementary Materials. Below 170 °C, hydrolysis was negligible, and the acid value remained low at 5.21 mg KOH/g close to the original value of 1.63 mg KOH/g. As the temperature increased to 170 °C, the acid value rose to 30.33 mg KOH/g, marking the onset of PET degradation. Upon further heating to 200 °C, the acid value reached a maximum of 91.13 mg KOH/g, corresponding to the highest PET conversion. FTIR analysis reveals distinct changes in the degradation liquid compared to pristine castor oil (Figure S3, Supplementary Materials). The carbonyl stretching band shifts from 1745 cm^−1^ to 1708 cm^−1^, accompanied by the appearance of a new band at 1466 cm^−1^, which is attributed to the -CH_2_- scissoring vibration of fatty acids. These spectral features confirm the hydrolysis of castor oil esters and the in situ formation of carboxylic acids.

These results indicate that castor oil alone does not depolymerize PET; rather, its hydrolysis generates fatty acids that act as catalysts. To further verify the catalytic role of these fatty acids, a control experiment was conducted using pure ricinoleic acid (the main component of castor oil) under the same conditions (3.0 g PET, 10.0 g water, 50.0 g ricinoleic acid, 200 °C, 10 h). The PET conversion reached only 42.03%, yielding a small amount of oligoester (Table S2). This confirms that while ricinoleic acid alone can indeed catalyze PET depolymerization, its efficiency is far lower than that of the water/castor oil system. Therefore, the high efficiency of the water/castor oil system cannot be attributed solely to the action of free fatty acids. Instead, it is more reasonably explained by the synergistic effect involving subcritical water, castor-oil-derived acidic species, glycerol, and PET-derived ethylene glycol.

To investigate the reaction kinetics of PET depolymerization in the water–castor oil system, To obtain a preliminary estimate of the apparent kinetic parameters, the depolymerization process was approximately described using a first order kinetic model as an empirical simplification [37,38], despite the complexity of the actual reaction system involving simultaneous hydrolysis, alcoholysis, and transesterification. The rate equation can be expressed as [39]:

(2) $$-\frac{dC_{\mathrm{PET}}}{\mathrm{dt}}\;=\;kC_{\mathrm{PET}}$$

where k is the rate constant and C(PET) is the concentration of PET at time t. The relationship between concentration and conversion is given by:

(3) $$\frac{C_{\mathrm{PET}}}{C_{\mathrm{PET},0}}\;=\;\frac{m_{1}}{m_{0}}\;=\;1-X$$

where $C_{\mathrm{PET},0}$ is the initial concentration of PET, and m_0_ and m_1_ are the initial and unreacted residual masses of PET, respectively. Substituting Equation (3) into Equation (2) yields:

(4) $$\frac{\mathrm{dX}}{\mathrm{dt}}=\;k(1-X)$$

Integration with respect to time gives:

(5) $$\ln\;(\frac{1}{1-X})\;=\;\mathrm{kt}$$

The depolymerization reactions were carried out at 170 °C, 175 °C, 180 °C, 185 °C, 190 °C and 200 °C for durations of 6, 8, 10, and 12 h, respectively, using a constant water-to-oil mass ratio of 1:5 under otherwise identical conditions. The PET degradation data at these temperatures were fitted to a first-order kinetic model, as shown in Figure 2c. The calculated rate constants k were 0.0335 h^−1^, 0.0735 h^−1^, 0.1984 h^−1^, 0.5039 h^−1^, 0.6281 h^−1^, and 0.6735 h^−1^ at 170 °C, 175 °C, 180 °C, 185 °C, 190 °C, and 200 °C, respectively, showing a pronounced increase with rising temperature. All fitted lines exhibited good linearity, with R^2^ values exceeding 0.98 (see Table S3 in the Supplementary Materials), supporting that the degradation follows first-order kinetics.

The activation energy (Ea) for the reaction was determined by fitting the temperature-dependent rate constants to the Arrhenius equation [40]:

(6) $$\ln\;k=-\frac{E_{a}}{R}\cdot \frac{1}{T}+\ln\;A$$

where k is the rate constant, A is the pre-exponential factor, Ea is the activation energy, R is the gas constant, and T is the absolute temperature in kelvin.

The resulting Arrhenius plot (Figure 2d) gave an apparent activation energy of 197.3 kJ/mol (R^2^ = 0.893). The moderate R^2^ value may partly reflect the complexity of the multicomponent system, where the apparent kinetics could be influenced by the initial activation stage and liquid–solid mass transfer, as reported in heterogeneous PET glycolysis [41]. Therefore, this Ea should be regarded as a rough estimate for comparative purposes, and its standard error was not calculated. It should also be noted that this value is averaged over the reaction course, as the continuous generation of fatty acids may alter the reaction environment during the process. To verify the reproducibility of the kinetic analysis, two independent runs were performed and the fitting results are provided in the Supplementary Materials (Figure S4, Tables S4 and S5).

### 3.3. Synergistic Effects and Process Optimization in PET Depolymerization

**Figure 3:**
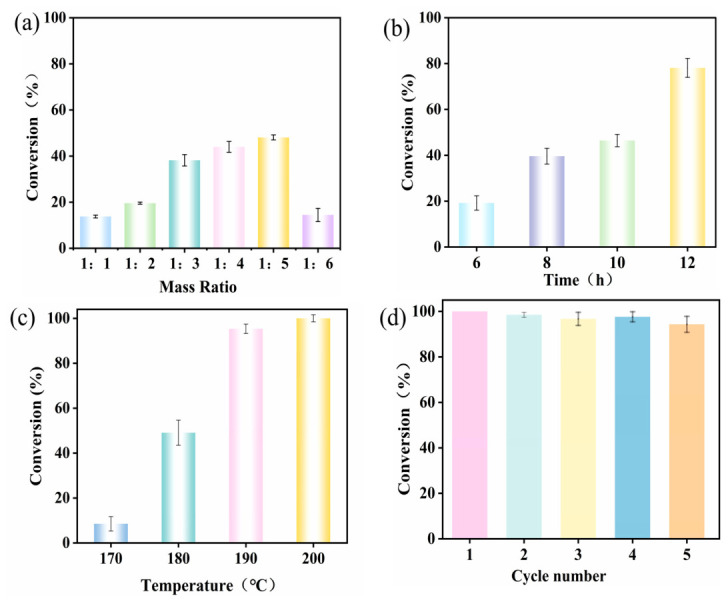
(**a**) PET conversion as a function of water-to-oil mass ratio (reaction conditions: 180 °C, 10 h). (**b**) Effect of reaction time on PET conversion (reaction conditions: 180 °C, water-to-oil mass ratio of 1:5). (**c**) Effect of temperature on PET conversion (reaction conditions: water-to-oil mass ratio of 1:5, 10 h). (**d**) Recyclability of the castor oil phase: PET conversion over five consecutive cycles under optimal conditions. The corresponding standard deviation values are provided in Tables S6–S9 in the Supplementary Materials.

The influence of the water-to-oil mass ratio on PET depolymerization was examined at 180 °C for 10 h. As shown in Figure 3a, PET conversion was highly dependent on the water content. In the absence of water, castor oil-despite its high hydroxyl value-exhibited limited reactivity due to steric hindrance from its long hydrophobic chains, which impeded nucleophilic attack on PET ester bonds and resulted in poor depolymerization. The introduction of water, particularly under subcritical conditions, markedly altered the reaction medium. The ionic product of water increased by several orders of magnitude compared to ambient conditions, producing abundant H_3_O^+^ and OH^−^ ions. These species promoted the partial hydrolysis of castor oil, generating ricinoleic acid and smaller, more accessible polyols such as glycerol. This process enhanced both the acidity of the system and the availability of nucleophilic hydroxyl groups, thereby improving PET depolymerization efficiency without an external catalyst.

An optimal water-to-oil mass ratio of 1:5 was identified, achieving 48.08% PET conversion. These results confirm a synergistic effect between castor oil and subcritical water, with the latter activating the bio-based oil to enable efficient self-catalytic depolymerization. Although trace amounts of TPA were detected at 180 °C, the yield was negligible and is not discussed further. Figure 3b illustrates the effect of reaction time on PET conversion at 180 °C and a water-to-oil mass ratio of 1:5. Prolonged reaction time allowed more opportunities for hydroxyl groups to attack ester bonds under the combined action of ricinoleic acid and H_3_O^+^, leading to a gradual increase in PET conversion, which reached a maximum of 78.14%. Figure 3c shows the influence of temperature on PET conversion at a fixed water-to-oil mass ratio of 1:5 and a reaction time of 10 h. Elevated temperatures promoted the formation of ricinoleic acid and increased both system acidity and hydroxyl reactivity, accelerating ester bond cleavage. Complete PET conversion was achieved at 200 °C.

To systematically evaluate key depolymerization parameters, an orthogonal experimental design was implemented with three factors-water-to-oil mass ratio (C), temperature (A), and time (B) each at four levels using an L16 (4^3^) orthogonal array. PET conversion values under each condition are summarized in Tables S10 and S11 (Supplementary Materials). Range analysis (Table S12) indicated that the influencing factors followed the order A > B > C, underscoring the dominant role of temperature. The optimal condition for PET conversion was identified as C_3_A_4_B_4_ (water-to-oil mass ratio 1:5, 200 °C, 12 h), highlighting the critical balance between water content and thermal activation for achieving high depolymerization efficiency. To determine the minimum reaction time required under the conditions of a water-to-oil mass ratio of 1:5 at 200 °C, it was found that a PET conversion of 100% could be achieved within 10 h (Figure S5, Supplementary Materials).

The reusability of the castor oil phase was evaluated under the optimal PET depolymerization condition (water/castor oil = 1:5, 200 °C, 10 h). As shown in Figure 3d, PET conversion remained above 94% over five consecutive cycles, indicating good recyclability of the castor oil phase. To further probe its recycling stability, the acid value and hydroxyl value after each cycle were measured (Table S13, Supplementary Materials). The acid value remained high throughout the five cycles (101.53–108.75 mg KOH/g), suggesting that the acidic environment responsible for PET depolymerization was largely retained. The hydroxyl value, although not strictly monotonic, also remained at an appreciable level (111.91–131.60 mg KOH/g), indicating the continued presence of hydroxyl-containing functionalities in the recycled medium. Moreover, the ^1^H NMR spectra of the recycled castor oil phase after the 1st and 5th cycles (Figure S6, Supplementary Materials) show that the characteristic signals at 12.1 ppm (-COOH), 8.05 ppm (Ar-H), and 5.37 ppm (-CH=CH-) were still retained, indicating that the key acidic, aromatic, and unsaturated structural features of the recycled oil phase were largely preserved during repeated use.

Analysis of variance (ANOVA) was performed on the PET conversion data to quantify the statistical significance of the three factors (Table S14). Temperature exhibited an extremely significant effect (F = 60.22, *p* < 0.01), while reaction time and water/castor oil ratio were not statistically significant within the investigated ranges, consistent with the range analysis. These results confirm that temperature is the dominant parameter controlling depolymerization.

Although the statistical optimum based on the orthogonal design was C_3_A_4_B_4_, further validation under the condition of a water-to-castor oil mass ratio of 1:5 at 200 °C showed that complete PET conversion could already be achieved within 10 h. Therefore, 1:5, 200 °C, and 10 h was selected as the practical optimum condition, considering both depolymerization efficiency and energy consumption. It should be noted that a complete mass balance was not established in this study due to the dynamic nature of the system, and is identified as a direction for future work.

### 3.4. Performance Optimization of PU Adhesives Using Recovered PET Products

**Figure 4:**
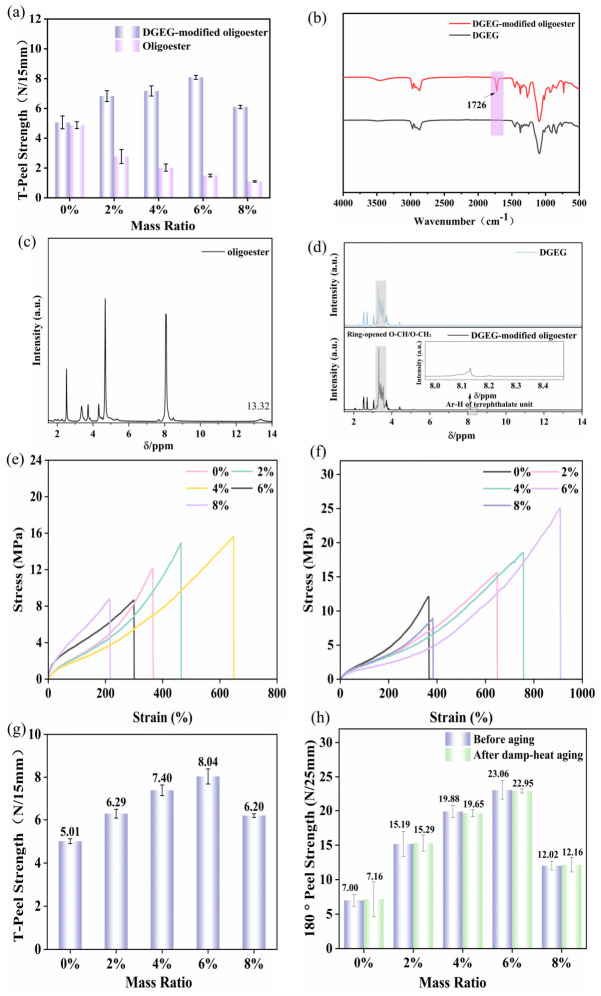
(**a**) T-peel strength of PET/Al laminate films incorporating oligoesters and DGEG-oligoester at different mass ratios. (**b**) FTIR spectra of DGEG, DGEG-modified oligoesters. (**c**) ^1^H NMR spectrum of the original oligoester. (**d**) ^1^H NMR spectra of DGEG and DGEG-modified oligoester. (**e**) Stress–strain curves of polyurethane films with different mass ratios of DGEG. (**f**) Stress–strain curves of polyurethane films with different mass ratios of DGEG-oligoesters. (**g**) T-peel strength of DGEG-modified oligoester-based polyurethane adhesives after damp-heat aging. (**h**) 180° peel strength of DGEG-modified oligoester-based polyurethane adhesives before and after damp-heat aging. The corresponding standard deviation values are provided in Tables S17–S21 in the Supplementary Materials.

For reference, the T-peel strength data of a castor-oil-based adhesive system are provided in the Supplementary Materials (Figure S7), since the present adhesive formulation was initially developed on the basis of that reference system. Within this formulation framework, a significant reduction in T-peel strength was observed with increasing loading of unmodified oligoester, as shown in Figure 4a. The T-peel strength decreased from 4.88 N/15 mm at 0% to 1.10 N/15 mm at 8%, which can be attributed to the poor compatibility of the oligoester with the polyurethane matrix and the adverse effect of residual carboxylic acid groups on polyurethane network formation. To address this limitation, the oligoester was chemically modified with DGEG through epoxy ring-opening esterification.

The success of the modification was confirmed by FTIR, ^1^H NMR, and functional-group analysis. In the FTIR spectra (Figure 4b), the DGEG-modified oligoester exhibits a characteristic carbonyl absorption at 1726 cm^−1^, consistent with ester formation after epoxy ring-opening. More direct structural evidence is provided by the ^1^H NMR spectra (Figure 4c,d). In the spectrum of the original oligoester (Figure 4c), a distinct signal at 13.32 ppm is assigned to the proton of free carboxyl groups. After DGEG modification, this signal disappears in the DGEG-oligoester spectrum, indicating substantial consumption of residual carboxylic acid groups. Meanwhile, the resonance signals in the 3.3–3.7 ppm region become markedly stronger, consistent with the formation of additional O-CH/O-CH_2_ structures after epoxy ring-opening. In addition, the aromatic proton signal at around 8.1 ppm remains detectable, confirming that PET-derived terephthalate units are retained in the modified product. Quantitative functional-group analysis (Table S15, Supplementary Materials) further supports the spectroscopic conclusions. The sharp decrease in acid value (from 126.29 to 3.62 mg KOH/g) and the concurrent increase in hydroxyl value (from 69.87 to 73.28 mg KOH/g) confirm the transformation of free carboxyl groups into hydroxyl-containing ester linkages through ring-opening esterification.

As shown in Figure 4a, the T-peel strength of unmodified oligoester adhesives decreased steadily with loading (from 4.88 to 1.10 N/15 mm), whereas DGEG-modified oligoester adhesives showed a unimodal trend, peaking at 6% loading (8.09 N/15 mm), which is notably higher than that of a previously reported PET/Al laminate adhesive system (5.8 N/15 mm) tested under the same standard [42], before declining at 8% (6.10 N/15 mm). Visual inspection of the peeled surfaces after the T-peel test (Figure S8) revealed adhesive residues on both the PET film and aluminum foil surfaces, suggesting mixed cohesive/interfacial failure rather than purely interfacial adhesive failure. Tensile strength (Figure 4f) and 180° peel strength before aging (Figure 4h) followed the same pattern, reaching maxima of 25.14 MPa and 23.06 N/25 mm, respectively, also at 6% loading. After damp-heat aging (85 °C/85% RH, 168 h), the unimodal trend persisted: the 6% formulation retained 8.04 N/15 mm in T-peel (0.62% loss) and 22.95 N/25 mm in 180° peel (0.47% loss), confirming excellent aging stability. TGA (Figure S9 and Table S16) showed that thermal stability first increased then decreased with modified oligoester content. The 6% D-O-PU formulation gave the best performance (T_5%_ = 309.38 °C, T_50%_ = 415.45 °C), outperforming both the unmodified control and the DGEG-only system across all loadings. At 8% loading, stability decreased, suggesting that excess modified oligoester compromises network uniformity. The observed thermal stability enhancement may be associated with the retained PET-derived aromatic rigid structures and increased hydroxyl functionality after DGEG modification. The consumption of residual carboxylic acid groups and formation of hydroxyl-containing ester linkages, confirmed by FTIR, ^1^H NMR, and functional-group analysis, indirectly support the improved reactivity of the modified oligoester in the PU system.

Overall, the adhesive performance can be explained by the chemical structure and functional-group changes of the PET-derived oligoesters. GPC confirmed the formation of low-molecular-weight oligoesters, while FTIR and ^1^H NMR verified the retained aromatic terephthalate units and residual carboxylic acid end groups. DSC and XRD further showed altered thermal and crystalline features compared with pristine PET, indicating the structural evolution of PET after depolymerization. The residual carboxylic acid groups in the unmodified oligoesters may have interfered with the polyurethane-forming reaction, leading to decreased peel strength. After DGEG modification, most carboxylic acid groups were consumed and hydroxyl-containing ester linkages were introduced, improving the reactivity of the oligoesters in the PU system. Consequently, at the optimal 6% loading, the retained aromatic structures and increased hydroxyl functionality contributed to improved mechanical strength and thermal resistance.

## 4. Conclusions

In summary, this study presents a self-catalytic strategy for PET depolymerization, wherein subcritical water enables castor oil to serve a dual role as both reaction medium and reactant. This process yields readily separable oligoesters. Compared with conventional BHET-oriented routes, these oligoesters possess higher molecular weights, allowing separation by centrifugation without crystallization. Through orthogonal experimental design, the effects of temperature, reaction time, and water-to-oil mass ratio on PET conversion were systematically evaluated, revealing that the influencing factors followed the order of temperature > time > water-to-oil mass ratio. Although the orthogonal design suggested an optimum combination of 200 °C, 12 h, and a water-to-oil mass ratio of 1:5, further validation showed that complete PET conversion could already be achieved within 10 h under these conditions. Under the practical optimum condition, the castor-oil phase retained good depolymerization performance over five consecutive cycles, with PET conversion remaining above 94%. Following DGEG esterification, the oligoesters could be directly employed as reactive components in polyurethane synthesis. The DGEG-modified oligoester at its optimal loading not only substantially outperformed the unmodified system in mechanical strength and thermal stability, but also demonstrated excellent retention of adhesion after accelerated aging. Overall, this work demonstrates a feasible upcycling route for converting PET into value added oligoesters for high-performance polyurethane adhesive applications.

## Data Availability

The original contributions presented in this study are included in the article/Supplementary Materials. Further inquiries can be directed to the corresponding authors.

## Supplementary Materials

The following supporting information can be downloaded at: https://www.mdpi.com/article/10.3390/ma19142977/s1, Figure S1: Analysis of molecular weight and distribution of PET and degradation products; Figure S2: SEM images of (a) Pristine PET (×430) and (b) the PET after degradation (×330); Figure S3: FTIR spectra of pristine castor oil and the degradation liquid; Figure S4: Kinetic analysis of PET depolymerization in the water–castor oil system: (a) first independent run—linear fitting of degradation rate constants at various temperatures; (b) second independent run—linear fitting of degradation rate constants at various temperatures; (c) Arrhenius plot derived from the temperature-dependent rate constants for determining the apparent activation energy; Figure S5: Effect of Reaction Time on PET Conversion (Water-to-Oil Ratio: 1:5, Temperature: 200 °C); Figure S6: Comparison of the ^1^H NMR spectra of the recycled castor oil phase after the 1st and 5th cycles; Figure S7: T-peel strength of PET/Al laminate films prepared using castor-oil-based polyurethane adhesives with different castor oil mass ratios; Figure S8: Photographs of the peeled surfaces after the T-peel test for the 6% DGEG-modified oligoester polyurethane adhesive; Figure S9: TG curves of polyurethane films containing (a) directly added DGEG and (b) DGEG-modified oligoesters at different mass ratios; Table S1: Acid value of castor oil at different temperatures; Table S2: Control experiment using PET, subcritical water, castor oil and ricinoleic acid under conditions comparable to the optimized depolymerization system; Table S3: Linear fitting equations and correlation coefficients (R^2^) of PET degradation rates at different temperatures in the water–castor oil system; Table S4: Linear fitting results of PET degradation rates at different temperatures in the water-castor oil system (first independent run); Table S5: Linear fitting equations and correlation coefficients (R^2^) of PET degradation rates at different temperatures in the water–castor oil system(second independent run); Table S6: Effect of water-to-castor oil mass ratio on PET conversion (180 °C, 10 h); Table S7: PET conversion at different reaction times (180 °C, 1:5); Table S8: Effect of temperature on PET conversion (1:5, 10 h); Table S9: PET conversion over five consecutive cycles of the castor-oil phase under the practical optimum condition (200 °C, 10 h, 1:5); Table S10: Orthogonal experimental factors and levels for PET depolymerization in the water–castor oil system; Table S11: Orthogonal experimental results of PET depolymerization: PET conversion; Table S12: Orthogonal experimental results of K, k, and range (R) values for PET conversion in the water–castor oil system; Table S13: Acid value and hydroxyl value of the recycled castor-oil phase after each cycle.; Table S14: Analysis of variance (ANOVA) for PET conversion based on the L16(4^3^) orthogonal design; Table S15: Hydroxyl and acid values of Oligoester, DGEG and DGEG-oligoester; Table S16: TG data of polyurethane films at different mass ratios; Table S17: T-peel strength of PET/Al laminate films containing oligoester at different mass ratios; Table S18: T-peel strength of PET/Al laminate films containing DGEG-modified oligoester at different mass ratios; Table S19: T-peel strength of polyurethane adhesives with different DGEG-modified oligoester contents after damp-heat aging; Table S20: 180° peel strength of adhesives with different DGEG-modified oligoester contents; Table S21: 180° peel strength of polyurethane adhesives with different DGEG-modified oligoester contents after damp-heat aging; Table S22: Formulation of DGEG-based polyurethane solutions; Table S23: Formulation of polyurethane adhesives containing DGEG-oligoester.
