# Supplementary material for: A Self-Catalytic Bio-Platform for Upcycling of PET Plastic into Oligoesters for Polyurethane Synthesis
Source: Materials (Basel). 2026 Jul 10;19(14):2977. doi: 10.3390/ma19142977 (PMC13412190; doi:10.3390/ma19142977)
Supplement: Supplementary file 1 [file materials-19-02977-s001.zip › materials-4409207-supplementary.pdf]

Supporting Information

A Self-Catalytic Bio-Platform for Upcycling of PET Plastic into Oligoesters For Polyurethane Synthesis

Anjie Qi[1], Yunjia Liang[2], Bingjie Ge[1], Guodong Jiang[*1], Shanglin Xiang, Dongyu Cai[*2]

[1] College of Materials Science & Engineering, Nanjing Tech University, 30 South PuZhu Road, Nanjing, Jiangsu, 211816, China

[2]School of Flexible Electronics (Future Technologies), Key Laboratory of Flexible Electronics and Institute of Advanced Materials, Nanjing Tech University, 30 South PuZhu Road, Nanjing, Jiangsu, 211816, China

*Corresponding author: iamdycai@njtech.edu.cn; gdjiang@njtech.edu.cn

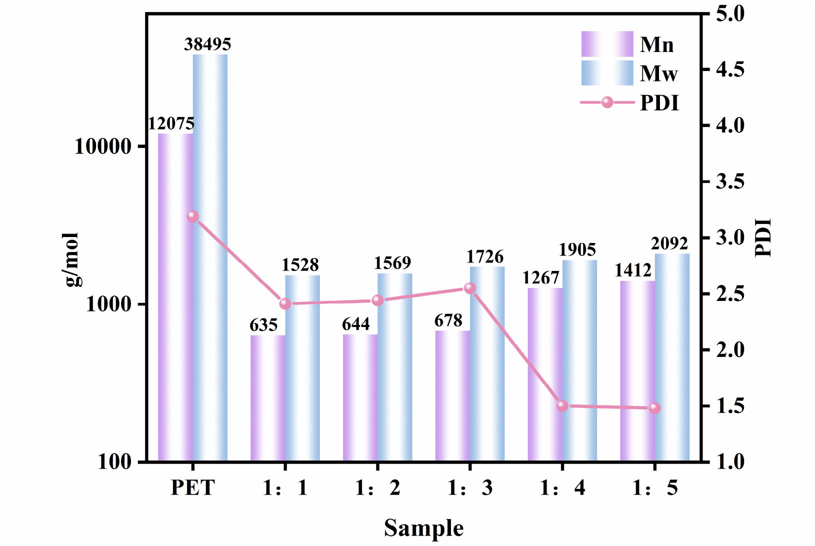

Figure S1. Analysis of molecular weight and distribution of PET and degradation products

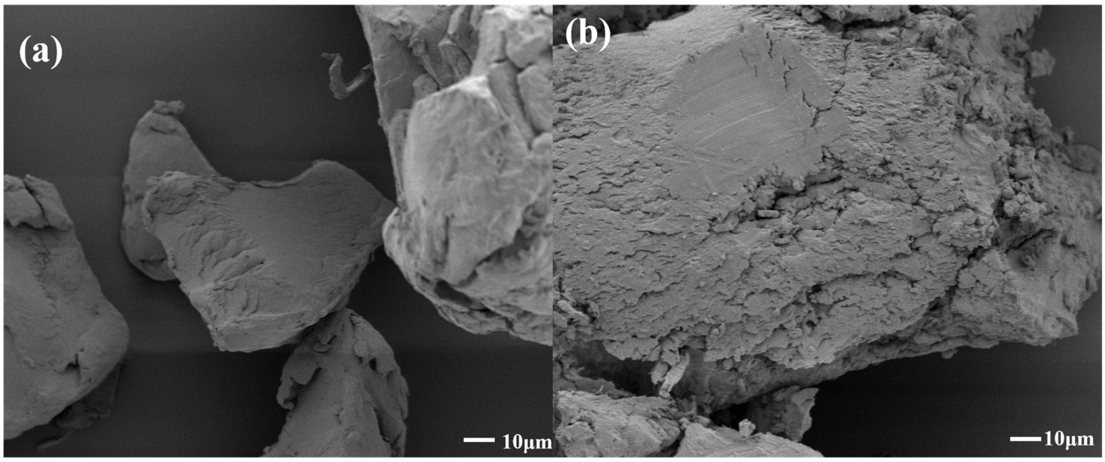

Figure S2. SEM images of (a) Pristine PET(×430) and (b) the PET after degradation(×330).

Table S1. Acid value of castor oil at different temperatures

Temperature (°C)	Acid value (mg KOH/g)
160	5.21±2.10
170	30.33±1.28
180	69.91±3.23
190	78.35±2.48
200	91.13±3.13

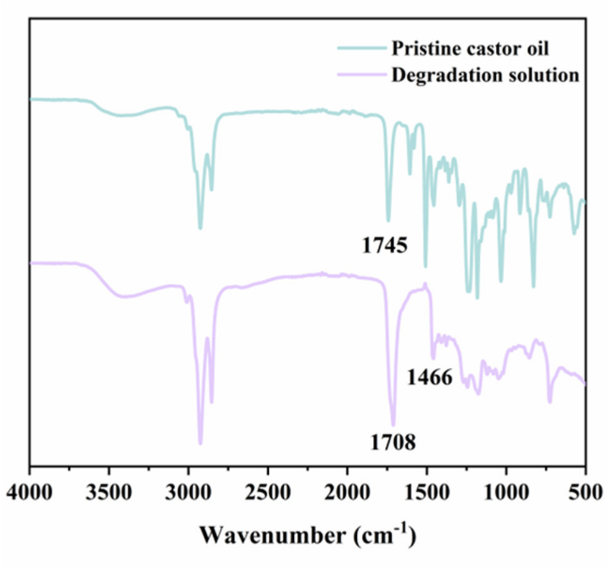

Figure S3. FTIR spectra of pristine castor oil and the degradation liquid.

Table S2. Control experiment using PET, subcritical water, castor oil and ricinoleic acid under conditions comparable to the optimized depolymerization system.

System	Temperature (°C)	Time (h)	PET conversion (%)	Products
PET + subcritical water + ricinoleic acid	200	10	42.03	Only a small oligoester
PET + water/castor oil system	200	10	100%	Oligoester and TPA-rich fraction
PET + castor oil	200	10	/	/

Table S3. Linear fitting equations and correlation coefficients (R^2) of PET degradation rates at different temperatures in the water–castor oil system

Temperature(℃)	Regression equation	Linear correlation coefficient
170	y=0.0335x-0.15376	0.99658
175	y=0.0735x-0.34567	0.99975
180	y=0.1984x-1.07434	0.98749
185	y=0.5039x-2.2716	0.99597
190	y=0.6281x-2.9714	0.99321
200	y=0.6735x-2.7502	0.99937

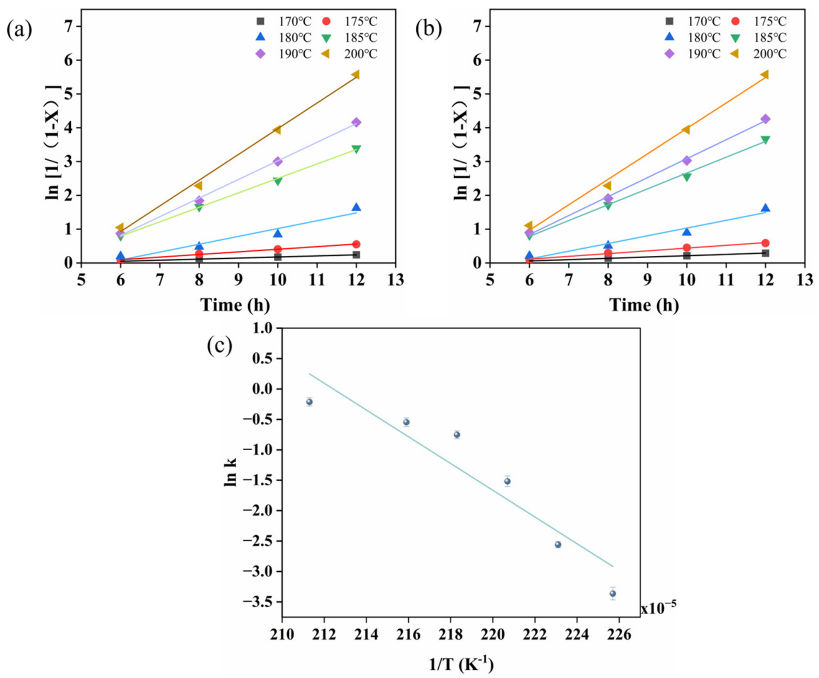

Figure S4. Kinetic analysis of PET depolymerization in the water–castor oil system: (a) first independent run - linear fitting of degradation rate constants at various temperatures; (b) second independent run - linear fitting of degradation rate constants at various temperatures; (c) Arrhenius plot derived from the temperature-dependent rate constants for determining the apparent activation energy.

For the Arrhenius analysis based on the second independent run, the linear fit of ln k versus 1/T gave the equation y = -21973.13985x + 46.67649, with a correlation coefficient of R^2 = 0.875.

Table S4. Linear fitting results of PET degradation rates at different temperatures in the water-castor oil system (first independent run)

Temperature(°C)	Regression equation	Linear correlation coefficient
170	y=0.0318x-0.1415	0.99358
175	y=0.0773x-0.36776	0.99272
180	y=0.2321x-1.30257	0.90938
185	y=0.4442x-1.8939	0.99913
190	y=0.5511x-2.4901	0.99583
200	y=0.7611x-3.6397	0.99449

Table S5. Linear fitting equations and correlation coefficients (R^2) of PET degradation rates at different temperatures in the water–castor oil system(second independent run)

Temperature(°C)	Regression equation	Linear correlation coefficient
170	y=0.0388x-0.17412	0.99682
175	y=0.08159x-0.3763	0.98747
180	y=0.22845x-1.2518	0.93783
185	y=0.4684x-2.024	0.99549
190	y=0.5606x-2.5217	0.99714
200	y=0.7526x-3.5466	0.99155

Table S6. Effect of water-to-castor oil mass ratio on PET conversion (180 °C, 10 h).

Water-to-castor oil mass ratio	PET conversion (%)
1:1	13.76±0.61
1:2	19.56±0.45
1:3	38.17±2.47
1:4	43.99±2.34
1:5	48.08±1.09
1:6	14.45±2.88

Table S7. PET conversion at different reaction times (180°C, 1:5).

Time (h)	PET conversion (%)
6	19.20±3.12
8	39.61±3.45
10	46.44±2.68
12	78.14±4.12

Table S8. Effect of temperature on PET conversion (1:5, 10 h).

Temperature (°C)	PET conversion (%)
170	8.53±3.2
180	49.08±5.06
190	95.34±2.1
200	100%

Table S9. PET conversion over five consecutive cycles of the castor-oil phase under the practical optimum condition (200 °C, 10 h, 1:5).

Cycle number	PET conversion (%)
1	100
2	98.52±1.10
3	96.74±2.91
4	97.65±2.20
5	94.34±3.53

Table S10. Orthogonal experimental factors and levels for PET depolymerization in the water–castor oil system

level	C	A	B
	Water-to-castor oil mass ratio	Temperature (°C)	Time (h)
1	1：3	170	6
2	1：4	180	8

Follow table S10. Orthogonal experimental factors and levels for PET depolymerization in the water–castor oil system

level	C	A	B
	Water-to-castor oil mass ratio	Temperature (℃)	Time (h)
3	1：5	190	10
4	1：6	200	12

Table S11. Orthogonal experimental results of PET depolymerization: PET conversion.

Run	C	A	B	PET Conversion (%)
1	1	1	1	12.41
2	1	2	2	29.92
3	1	3	3	100
4	1	4	4	100
5	2	1	2	19.24
6	2	2	1	39.09
7	2	3	4	100
8	2	4	3	100
9	3	1	3	31.94
10	3	2	4	78.14
11	3	3	1	88.15
12	3	4	2	100
13	4	1	4	21.35
14	4	2	3	44.84
15	4	3	2	93.24
16	4	4	1	100

Table S12. Orthogonal experimental results of K, k, and range (R) values for PET conversion in the water–castor oil system

	C	A	B
K1	242.33	84.94	239.65
K2	258.33	171.32	242.40
K3	298.23	381.39	256.11
K4	238.76	400	299.49
k1	60.58	21.24	59.91
k2	64.58	42.83	60.60
k3	74.56	93.35	64.03
k4	59.69	100	74.87
R	14.87	78.76	14.96

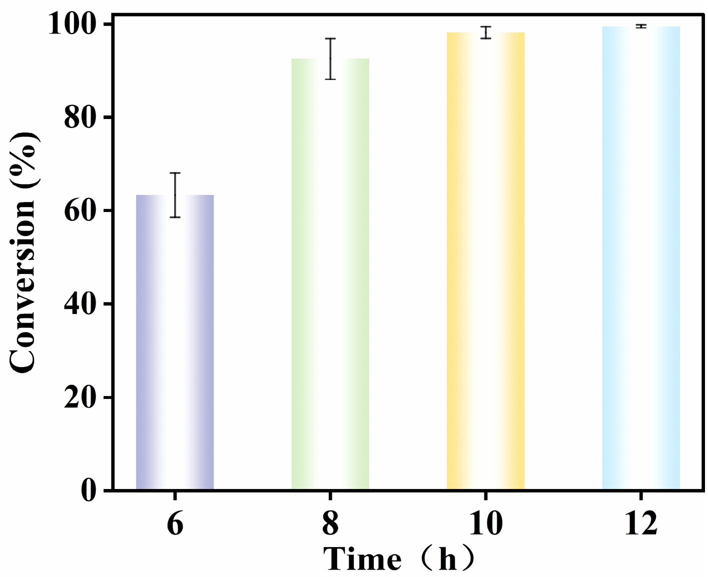

Figure S5. Effect of Reaction Time on PET Conversion (Water-to-Oil Ratio: 1:5, Temperature: 200 °C)

Table S13. Acid value and hydroxyl value of the recycled castor-oil phase after each cycle.

Cycle number	Acid value (mg KOH/g)	Hydroxyl value(mg KOH/g)
1	101.53±1.11	131.60±1.58
2	106.78±0.36	130.83±0.60
3	108.33±0.41	127.58±0.76
4	108.75±0.41	128.27±0.77
5	102.91±0.12	111.91±0.19

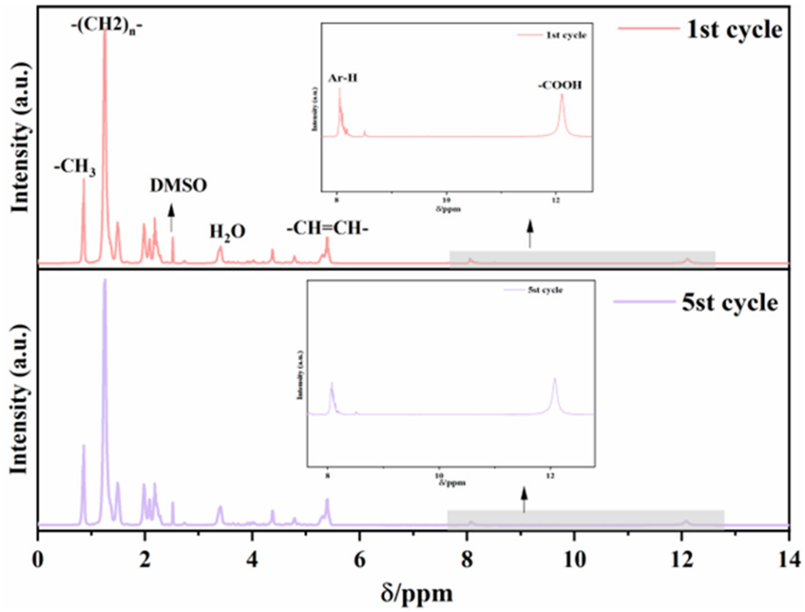

Figure S6. Comparison of the ^{1}H NMR spectra of the recycled castor oil phase after the 1st and 5th cycles.

Table S14. Analysis of variance (ANOVA) for PET conversion based on the L16(4^3) orthogonal design

Factor	Df	SS	MS	F	P	Significance
Temperature (A)	3	17380.75	5793.58	60.22	0.00007	Highly significant
Time (B)	3	620.25	206.75	2.15	0.1953	Not significant

Follow table S14. Analysis of variance (ANOVA) for PET conversion based on the L16(4^3) orthogonal design

Factor	Df	SS	MS	F	P	Significance
Water-to-castor oil mass ratio (C)	3	423.24	141.08	1.47	0.3150	Not significant
Residual	6	577.19	96.20	/	/	/
Total	15	19001.44	/	/	/	/

Note: df = degrees of freedom; SS = sum of squares; MS = mean square. The residual term contains both experimental error and unresolved interaction effects.

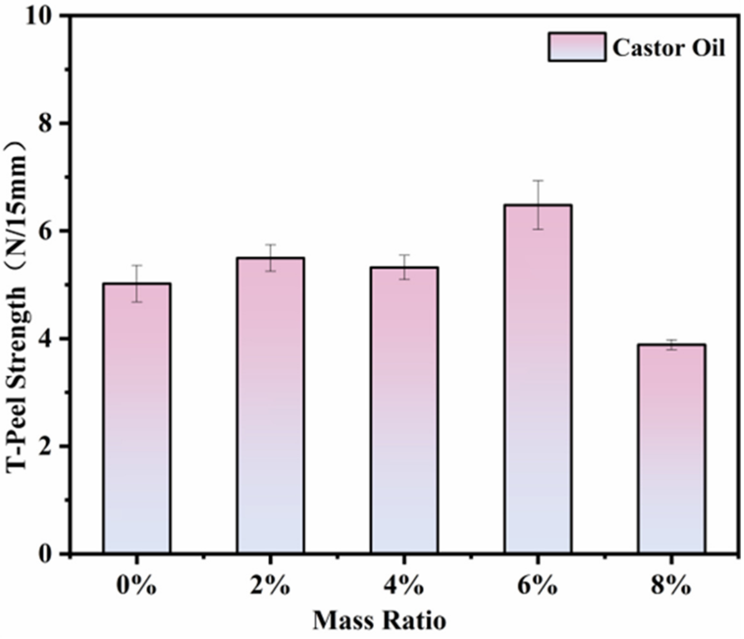

Figure S7. T-peel strength of PET/Al laminate films prepared using castor-oil-based polyurethane adhesives with different castor oil mass ratios.

Table S15. Hydroxyl and acid values of Oligoester ,DGEG and DGEG-oligoester.

Sample	HV (mgKOH/g)	AV (mgKOH/g)
Oligoester	69.87	126.29
DGEG	82.09	1.74
DGEG-oligoester	73.28	3.62

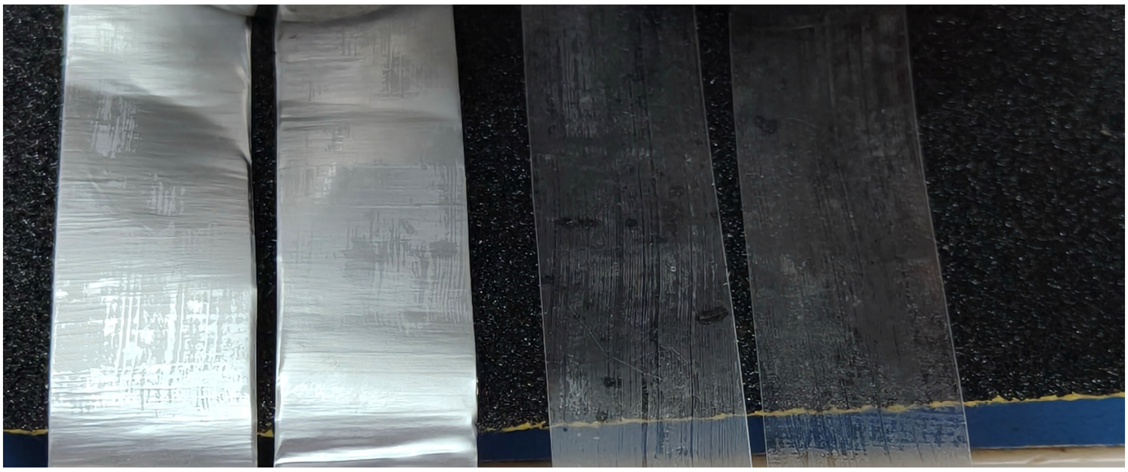

Figure S8. Photographs of the peeled surfaces after the T-peel test for the 6% DGEG-modified oligoester polyurethane adhesive.

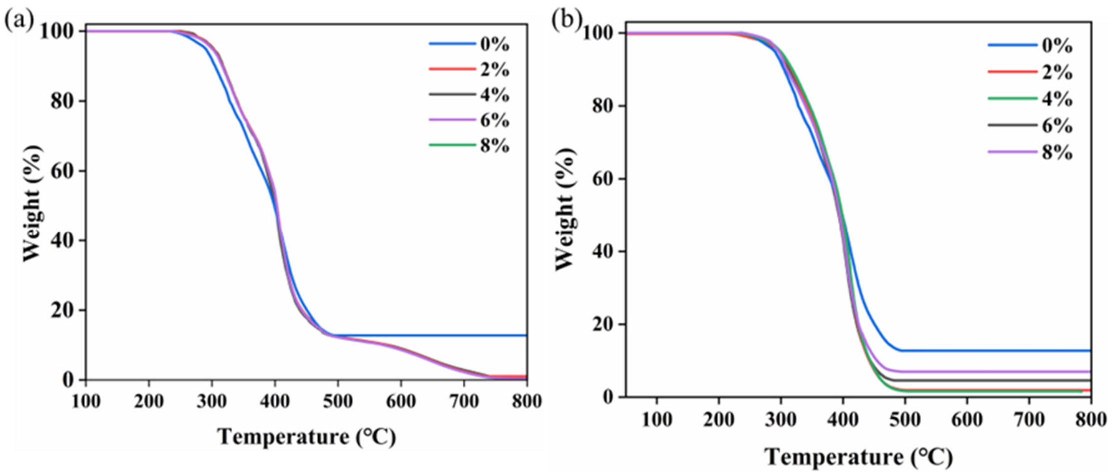

Figure S9. TG curves of polyurethane films containing (a) directly added DGEG and (b) DGEG-modified oligoesters at different mass ratios.

Table S16. TG data of polyurethane films at different mass ratios.

Sample	$T_{5\%}$ (°C)	$T_{50\%}$ (°C)	Residual mass (%)
0%D-PU	289.44	398.99	12.75
2%D-PU	301.29	400.14	1.05
4%D-PU	302.21	402.99	0.14
6%D-PU	304.42	407.34	0.15
8%D-PU	300.11	400.63	0.26
0%D-O-PU	289.86	398.95	12.75
2%D-O-PU	304.10	411.68	7.12
4%D-O-PU	304.99	406.45	4.19
6%D-O-PU	309.38	415.45	11.04
8%D-O-PU	293.19	401.02	4.75

Note: X%D-PU denotes polyurethane films containing X wt.% of directly added DGEG, while X%D-O-PU denotes polyurethane films containing X wt.% of DGEG-modified oligoester, where X represents the mass percentage of the corresponding additive.

Table S17. T-peel strength of PET/Al laminate films containing oligoester at different mass ratios.

Oligoester content (%)	T-peel strength (N/15 mm)
0	4.88±0.33
2	2.77±0.47
4	2.03±0.24
6	1.50±0.09
8	1.10±0.05

Table S18. T-peel strength of PET/Al laminate films containing DGEG-modified oligoester at different mass ratios.

DGEG-modified oligoester content (%)	T-peel strength (N/15 mm)
0	5.07±0.43
2	6.83±0.37
4	7.18±0.34
6	8.09±0.14
8	6.10±0.11

Table S19. T-peel strength of polyurethane adhesives with different DGEG-modified oligoester contents after damp-heat aging.

DGEG-modified oligoester content (%)	T-peel strength (N/15 mm)
0	5.01±0.11
2	6.29±0.21
4	7.40±0.25
6	8.04±0.35
8	6.20±0.08

Table S20. 180° peel strength of adhesives with different DGEG-modified oligoester contents.

DGEG-modified oligoester content (%)	180 ° Peel Strength (N/25 mm)
0	7.00±0.84
2	15.19±1.83
4	19.88±0.88
6	23.06±1.39
8	12.02±0.62

Table S21. 180° peel strength of polyurethane adhesives with different DGEG-modified oligoester contents after damp-heat aging.

DGEG-modified oligoester content (%)	180 ° Peel Strength(N/25mm)
0	7.16±2.51
2	15.29±1.17
4	19.65±0.52

Follw table S21. 180° peel strength of polyurethane adhesives with different DGEG-modified oligoester contents after damp-heat aging.

DGEG-modified oligoester content (%)	180 ° Peel Strength(N/25mm)
6	22.95±0.31
8	12.16±1.04

Table S22. Formulation of DGEG-based polyurethane solutions

Sample	PCL -2000 (g)	L- MDI (g)	BDO (g)	DGEG (g)	EA (g)
1	40	7	1.49	0	48.49
2	40	7	1.25	2	50.25
3	40	7	1.00	4	52
4	40	7	0.75	6	53.75
5	40	7	0.5	8	55.5

Table S23. Formulation of polyurethane adhesives containing DGEG-oligoester

Sample	PCL -2000 (g)	L-MDI (g)	BDO (g)	DGEG-Oligoester (g)	EA (g)
1	40	7	1.49	0	48.49
2	40	7	1.25	2	50.25
3	40	7	1.00	4	52
4	40	7	0.75	6	53.75
5	40	7	0.5	8	55.5
